# Draft Genome Sequences of Nine Strains of Brochothrix thermosphacta, Carnobacterium divergens, Lactobacillus algidus, Lactobacillus fuchuensis, Lactococcus piscium, Leuconostoc gelidum subsp. gasicomitatum, Pseudomonas lundensis, and Weissella viridescens, a Collection of Psychrotrophic Species Involved in Meat and Seafood Spoilage

**DOI:** 10.1128/genomeA.00479-18

**Published:** 2018-06-14

**Authors:** Simon Poirier, Gwendoline Coeuret, Marie-Christine Champomier-Vergès, Stéphane Chaillou

**Affiliations:** a MICALIS Institute, INRA, AgroParisTech, Université Paris-Saclay, Jouy-en-Josas, France

## Abstract

In this study, we present the draft genome sequences of nine strains from various psychrotrophic species identified in meat products and being recognized as important emerging food spoilers. Many of these species have only one or few strains being sequenced, and this work will contribute to the improvement of the overall genomic knowledge about them.

## GENOME ANNOUNCEMENT

The bacterial communities involved in meat and seafood spoilage during storage at low temperature (0 to 8°C) include a wide range of species (1). Some of these food spoilers were characterized during the 1970s, such as Brochothrix thermosphacta or Carnobacterium divergens, but others were identified very recently, and their roles in food spoilage were only confirmed because noncultural 16S rRNA gene metagenetic analysis and/or plating on culture medium at low temperatures were performed. Most of these spoilers (old or emerging) thriving in cold environments usually share a low level of genomic identification, with only few strains being sequenced. This lack of genomic reference makes food spoilage metagenomic analysis difficult to perform. Here, we present the draft sequencing of several strains isolated from spoiled meat (2), including Brochothrix thermosphacta strain 160x8, representing an old spoiler isolated in 1981 from a horse steak; Lactococcus piscium strains CMTALT02 and CMTALT17, isolated from spoiled beef carpaccio and representing two new members from this psychrotrophic species that is ubiquitous in meat and seafood products (3); Leuconostoc gelidum subsp. gasicomitatum strain MFPA44A1401 and Lactobacillus algidus strain CMTALT10, representing the paradigm of emerging highly psychrotrophic spoilers that are tedious to cultivate (4–6); Lactobacillus fuchuensis strain MFPC41A2801, representing a poorly known member of the Lactobacillus sakei phylogenetic clade and which is a species that is often subdominant in meat bacterial ecosystems but whose role as a spoiler remains to be deciphered (7); Carnobacterium divergens strain MFPA43A1405, representing a species with high survival fitness in many meat-processing environments; Pseudomonas lundensis strain MFPA15A1205, representing a species closely related to another food spoiler, Pseudomonas fragi, but which appears to be highly prevalent in beef stored under a vacuum and which harbors strong biofilm formation capacity on meat (8); and finally, Weissella viridescens strain MFPC16A2805, representing the paradigm of a sporadic spoiler (9) involved in visual spoilage (green iridescence on meat slices and package swelling due to CO_2_ production). This last strain also was revealed to possess one of the smallest genomes (∼1 Mb) ever sequenced for a lactic acid bacterium.

Details of the draft genomes are given in Table 1. Whole-genome sequencing of these strains was carried out by Eurofins MWG Operon Laboratories (Ebersberg, Germany) using Illumina MiSeq 2 × 150-bp paired-end libraries. Reads were assembled *de novo* by the Velvet software (10). All contigs were aligned against a relevant complete genome of closely related strains or species using progressiveMauve (11), and annotation was performed with the MicroScope platform (12).

**TABLE 1:** Overview of the draft genome assemblies from nine strains of emerging meat spoilers

Species	Source, yr	Original strain name (synonym)	Collection name	Genetic element	No. of contigs	Size (bp)	Coverage (×)	No. of CDS^*a*^	BioProject no.	Assembly accession no.
Brochothrix thermosphacta	Horse meat, 1981	J160x8 (HO01)	CIP 110942	Chromosome	32	2,443,096	70	2,473	PRJEB21917	OBMV01000001 to OBMV01000032
				Plasmid pJOUY160xA1	1	44,938	85	48		OBMV01000033
Carnobacterium divergens	Beef carpaccio, 2009	MFPA43A1405	CIP 110938	Chromosome	31	2,702,442	62	2,686	PRJEB21906	OGVA01000001 to OGVA01000031
Lactobacillus algidus	Beef carpaccio, 2013	CMTALT10	CIP 110929	Chromosome	20	1,624,384	56	1,582	PRJEB21909	OBKY01000001 to OBKY01000020
				Plasmid pCMTALT10B	4	13,563	302	19		OBKY01000021 to OBKY01000024
Lactobacillus fuchuensis	Beef carpaccio, 2009	MFPC41A2801	CIP 110928	Chromosome	51	2,045,293	65	1,977	PRJEB21913	OGVC01000001 to OGVC01000051
Lactococcus piscium	Beef carpaccio, 2013	CMTALT02	CIP 110936	Chromosome	35	2,154,856	63	2,177	PRJEB21910	OGVB01000001 to OGVB01000035
Lactococcus piscium	Beef carpaccio, 2013	CMTALT17	CIP 110937	Chromosome	68	2,271,863	63	2,307	PRJEB21911	OBKP01000001 to OBKP01000068
Leuconostoc gasicomitatum	Beef carpaccio, 2009	MFPA44A1401	CIP 110927	Chromosome	50	1,895,812	35	1,960	PRJEB21908	OBMW01000001 to OBMW01000050
Pseudomonas lundensis	Beef carpaccio, 2009	MFPA15A1205	CIP 110941	Chromosome	56	5,019,826	52	4,871	PRJEB21912	OBKZ01000001 to OBKZ01000056
Weissella viridescens	Beef carpaccio, 2009	MFPC16A2805	CIP 110940	Chromosome	13	1,012,612	149	1,024	PRJEB22329	OBHM01000001 to OBHM01000013
				Plasmid pMFPC16A2805B	1	16,484	344	15		OBHM01000014
				Plasmid pMFPC16A2805C	1	11,586	507	12		OBHM01000015

a CDS, coding sequences.

### Accession number(s).

The sequence data have been deposited in DDBJ/ENA/GenBank under the accession numbers cited in Table 1.
